# The protective effects and mechanisms of rosmarinic acid against *Pseudomonas aeruginosa* infection in *Caenorhabditis elegans*

**DOI:** 10.3389/fphar.2025.1701885

**Published:** 2025-12-02

**Authors:** Ying Zhang, Chengjie Shu, Zhuohang Li, Man Qu, Chunwu Wang, Shihui Nie, Qijiang Xu, Shunlin Gu, Mingjie Fan, Baojun Shi, Lei Fu, Fenglun Zhang

**Affiliations:** 1 School of Pharmacy, Nanjing University of Chinese Medicine, Nanjing, China; 2 Nanjing Institute for Comprehensive Utilization of Wild Plants, Nanjing, China; 3 School of Basic Medical Sciences and School of Public Health, Faculty of Medicine, Yangzhou University, Yangzhou, China; 4 Xinjiang Agricultural Technology Promotion Station, Urumqi, China; 5 Xinjiang Academy of Agricultural Sciences, Urumqi, China; 6 Yuepuhu Cumin Science and Technology Institute Co., Ltd., Kashgar, China; 7 Xinjiang Yuanda Green Agriculture Development Co., Ltd., Kashgar, China

**Keywords:** rosmarinic acid, *Pseudomonas aeruginosa*, anti-infection, bacterial infection, mitochondria, *Caenorhabditis elegans*

## Abstract

**Introduction:**

Pseudomonas aeruginosa poses a significant risk to both the environment and organisms. Although the natural compound rosmarinic acid (RA) has demonstrated considerable antibacterial properties, its efficacy in combating P. aeruginosa infection remains vague.

**Methods:**

Using Caenorhabditis elegans as an infection model, we evaluated the damage induced by PA14, examining indicators including lifespan, locomotor behavior, pharyngeal pumping, excretion cycle, lipofuscin, reactive oxygen species (ROS), as well as mitochondrial membrane potential (MMP) and the expression of mitochondrial-related genes mev-1 and gas-1.

**Results:**

PA14 infection had a diverse effect on C. elegans, including reduced lifespan, impaired behavioral functions (diminished head thrashing frequency, body bending frequency, and pharyngeal pumping frequency, as well as a prolonged excretion cycle), increased lipofuscin and ROS accumulation, and mitochondrial dysfunction (lower MMP and ATP content). RA treatment at concentrations of 100-300 mg/L dose-dependently reduced the number of PA14 colonies in the nematodes, prolonged the lifespan of the infected nematodes, improved behavioral impairments, decreased lipofuscin and ROS accumulation, and alleviated oxidative stress. Mechanistically, RA upregulated the expression profiles of the mev-1 and gas-1 mitochondrial-related genes involved in mitochondrial complex assembly and function. This enhanced MMP and ATP synthesis and mitigated PA14-induced mitochondrial dysfunction.

**Discussion:**

These results indicated that RA protected C. elegans from PA14 infection via its antioxidant properties and its ability to improve mitochondrial function, highlighting its potential as a natural compound for addressing PA14 contamination.

## 1 Introduction

*Pseudomonas aeruginosa* functions as a Gram-negative pathogenic bacterium widely distributed in the natural environment, including soil, water, air, and various moist surfaces (Velonakis et al., 2014). It secretes a variety of extracellular enzymes and toxins, which disrupt the balance of the surrounding microbial community, inhibit the growth of beneficial microorganisms, and affect the material cycle and energy flow in the ecosystem (Gellatly and Hancock, 2013). In soil, *P. aeruginosa* reduces the soil microbial diversity, impairs plant growth and development, and disrupts ecological balance (Licea-Herrera et al., 2024; Thomas and Sekhar, 2016). Massive *P. aeruginosa* proliferation in water bodies may cause water eutrophication, while secreted metabolites, such as pyocyanin, are toxic to aquatic organisms, threatening their survival and the marine ecosystem (Mena and Gerba, 2009). *P. aeruginosa* can cause chronic, acute, and refractory infections in mammals and invertebrates, and it is a key pathogen inducing pneumonia. In cystic fibrosis mice, *P. aeruginosa* exacerbates the deterioration of lung function and is one of the main factors contributing to their death (Matsumoto et al., 2016; Van’t Wout et al., 2015).

*Caenorhabditis elegans* is commonly utilized to investigate the mechanisms underlying pathogenic infection. This transparent nematode has a short developmental cycle, allowing the direct observation of pathogenic colonization, dissemination, and pathological changes in host tissues *in vivo* (Kumar et al., 2020). Additionally, the phenotypic changes after infection, such as death, developmental arrest, and intestinal lesions, can be monitored in a short timeframe. Advanced genetic techniques, such as gene knockout, transgenesis, and RNA interference (RNAi), are typically used to analyze host gene function during infection. As a simplified host model, *C. elegans* enables the intuitive elucidation of host–pathogen interaction mechanisms (Balla and Troemel, 2013). *C. elegans* exhibits stress responses after infection, allowing clarification of the infection mechanisms via factors such as heat shock protein expression and oxidative stress pathway activation (Servello and Apfeld, 2020). For example, infecting *C. elegans* with *Klebsiella pneumoniae* upregulates the *hsp-16* gene (a heat shock protein gene), enhancing the survival rate by repairing protein damage, which is consistent with the heat shock response mechanism in human infections (Kamaladevi and Balamurugan, 2017; Schmauder et al., 2022). The innate immune pathways of *C. elegans* represent a “simplified version” of the immune mechanisms of more complex organisms (Ermolaeva and Schumacher, 2014). For instance, the p38 MAPK pathway is highly conserved with the p38-mediated inflammatory factor release mechanism in human macrophages. Its activation induces the expression of antimicrobial peptides, which is crucial for *C. elegans* resistance to Gram-negative bacterial infections (Kim et al., 2002; Yang et al., 2022). Signaling pathways such as the insulin-like pathway regulate *C. elegans* aging and participate in infection responses, linking aging with infection susceptibility, regulating aging-related genes, enhancing antimicrobial peptide expression, and prolonging survival time after infection (Venz et al., 2021; Lee and Lee, 2022).

Rosmarinic acid (RA) is a natural phenolic acid compound widely distributed in plant families such as *Apiaceae*, *Lamiaceae*, *Boraginaceae*, and *Cucurbitaceae* and in *Cuminum cyminum* L. (Petersen and Simmonds, 2003). Cumin is a significant natural source of the potent antioxidant RA, which may be more concentrated in immature cumin seeds due to higher metabolic activity in response to environmental challenges such as ultraviolet radiation and microbial invasion (Bettaieb, I et al., 2010; Bettaieb Rebey et al., 2011). In addition to cumin, RA is also found in plants such as rosemary, mint, and perilla. Recent years have seen significant advancement in research on the pharmacological effects of RA. Studies have shown that RA displays various biological activities, including antibacterial, anti-inflammatory, antioxidant, antitumor, and neuroprotective properties (Guan et al., 2022). It can inhibit various bacteria, fungi, and viruses, while its action mechanisms may be related to the disruption of microbial cell membrane structures and the inhibition of nucleic acid and protein synthesis. Furthermore, the antioxidant ability of RA allows it to scavenge free radicals in the body and reduce oxidative stress-induced cell damage, showing potential for preventing and treating various diseases (Gui et al., 2021). Current research on the pharmacological effects of RA focuses on molecular mechanisms, providing a solid theoretical basis for its clinical application.

PA14 is a strain of *P. aeruginosa* and is used to establish multi-host pathogenic models. Owing to its high pathogenicity, complete genome, and ease of cultivation, PA14 has become a classic model strain for studying virulence factors, quorum sensing (QS) systems, antimicrobial screening, and host–pathogen interactions of *P. aeruginosa* (Grace et al., 2025). PA14 is highly compatible with the *C. elegans* model and is one of the most widely used strains in *C. elegans* pathogenic models. It has been extensively applied in researching bacterial virulence, host immunity, behavioral learning, and drug screening (Tan et al., 1999). Feinbaum et al. (2012) performed genome-wide screening of virulence genes using the *C. elegans* infection model and validated the compatibility and reliability of PA14 in this model. In summary, this study utilizes PA14 as the pathogen in the infection model and *C. elegans* as the host organism. PA14 exhibits strong pathogenicity, while its biological characteristics are highly compatible with the advantages of the *C. elegans* model, enabling efficient simulation of the key processes involved in the “host–pathogen interaction” (Grace et al., 2022; Wang et al., 2021). Although RA is a natural compound with multiple pharmacological activities, its ability to inhibit PA14 infection and its underlying mechanism remain unclear. This study explores the protective effect and mechanism of RA against *P. aeruginosa* infection in *C. elegans*, providing a theoretical basis and experimental foundation for the development of natural compounds against *P. aeruginosa* infection.

## 2 Materials and methods

### 2.1 Husbandry of animals

Wild-type N2 *C. elegans* were cultured on nematode growth medium (NGM) plates and fed with *E. coli* OP50 to maintain worm development. Gravid nematodes were treated with a lysis solution (a 1:1 volume mixture of 10% NaClO and 1 M NaOH) for nematode synchronization. The collected eggs were placed on new NGM plates and cultured to the young adult stage for experimental use (Hunt, 2017).

### 2.2 *C. elegans* infection with PA14

PA14 was cultured in Luria–Bertani (LB) broth and then inoculated onto modified NGM killing plates containing 0.35% peptone. The cultures were incubated at 37 °C for 24 h, followed by further incubation at 25 °C for another 24 h. The synchronized young adults were transferred to NGM plates containing PA14, at 50 worms per plate, and incubated at a constant temperature of 20 °C (Yuan et al., 2024).

### 2.3 Rosmarinic acid exposure

Post-PA14 infection, the synchronized L4-stage worms were kept under continuous exposure to various RA concentrations (100 mg/L, 200 mg/L, and 300 mg/L) over a 24-h period.

### 2.4 Colony count determination

After synchronization, the worms were cultured in groups with PA14 and different RA concentrations. After repeated washing with M9 buffer to remove the bacteria adhering to the body surfaces, 50 worms from each group were transferred to a grinder to disrupt the nematode body wall and release internal bacteria. The bacterial suspension was serially diluted, spread on LB culture plates, and incubated at 37 °C, after which the number of colonies was determined (Zhang, L. et al., 2022). Three repetitions of the experiment were performed.

### 2.5 Lifespan assay

Young adult nematodes exposed to the drug or infected with PA14 were placed on NGM agar plates seeded with OP50 using a platinum wire needle. For each group, 30 worms were selected in duplicate, and the cultures were maintained at a constant temperature of 20 °C. The lifespan measurements began on the day the worms were placed on the plates. Worms were deemed dead in instances where they did not move for 5 s after coming into contact with the platinum wire needle (Park et al., 2017). The survival rates of the nematodes were recorded daily until all the worms in a group were deceased. The point at which half of the nematodes in a group had died was designated as the median lifespan.

### 2.6 Behavioral analysis

A microscope was used to observe the head-waving and body-bending frequencies. A successful head wave was characterized by a change in the head movement direction that aligned with the orientation of the body, while a body bend was defined as the sinusoidal movement of the nematode body along its S-shaped midline (Zhang, H. et al., 2022). The body-bending and head-thrashing frequencies were recorded for 1 min, with the results expressed as bends and thrashes per minute. Twenty worms from each group were observed, and three repetitions of the experiment were performed.

The pharyngeal pumping frequency was observed under a microscope. Successful pharyngeal pumping was defined as a single contraction during the process of swallowing food (Keith et al., 2014). The pumping rate was recorded for 20 s. The excretion cycle was defined as the time required for the nematode body wall muscles to contract and excrete until the next contraction (Wu et al., 2013). Twenty worms from each group were observed, and three repetitions of the experiment were performed.

### 2.7 Lipofuscin level determination

Synchronized N2 worms were cultured with the respective treatments for 24 h according to their experimental groups and then washed with M9 buffer. Then, 500 μL of 4% paraformaldehyde was added to fix the worms for 20 min. An appropriate number of worms were transferred to 2% agarose pads and imaged under the DAPI channel at a wavelength of 525 nm. Images were analyzed using ImageJ software after normalization with autofluorescence, and the mean pixel density was calculated to assess the level of lipofuscin accumulation in the worms (Shen et al., 2021). Twenty worms from each group were assessed, and three repetitions of the experiment were performed.

### 2.8 Reactive oxygen species level measurement

The fluorescent probe 2′,7′-dichlorodihydrofluorescein diacetate (DCFH-DA) method was used to detect the reactive oxygen species (ROS) levels in the *C. elegans* (Lin et al., 2019; Moliner et al., 2020). Worms from the different treatment groups were placed in 96-well plates, after which a 50 μL M9 buffer and 50 μL DCFH-DA mixture was added to each well, followed by incubation for 2 h at 37 °C in the dark while shaking at 100 rpm. After staining, the probe was removed by washing with M9 buffer. Worms from each group were transferred to 2% agarose pads and imaged under the FITC green fluorescent channel, with an excitation wavelength of 485 nm and an emission wavelength of 520 nm. Images were analyzed using ImageJ software after normalization with autofluorescence. Twenty worms from each group were assessed, and three repetitions of the experiment were performed.

### 2.9 Mitochondrial function determination

The JC-1 probe method functioned to ascertain the mitochondrial membrane potential in the worms (Zhang et al., 2022a). JC-1 assumes a monomeric state under conditions of low membrane potential, which corresponds to green fluorescence with an emission wavelength of 525 nm (λem = 525 nm). In contrast, at high membrane potential, it assembles into aggregates, associated with red fluorescence emitting at 590 nm (λem = 590 nm), with the membrane potential reflected by the red-to-green fluorescence ratio. The JC-1 stock solution was diluted to 5–10 μM with M9 buffer and preheated at 37 °C. Worms from the different groups were resuspended in the JC-1 working solution, incubated in the dark at 20 °C for 20–30 min, placed on 2% agarose pads with 10 mM NaN_3_ (anesthetic), and imaged using a fluorescence microscope. Processing of the images was carried out using ImageJ to calculate the red-to-green fluorescence intensity ratio (red/green).

Worms from the different treatment groups were rinsed using M9 buffer and transferred to a grinder. They were ground manually in an ice water bath, after which the homogenate was centrifuged at 4500 r/min for 10 min. The supernatant was harvested, and the ATP content was determined according to the instructions of the corresponding kits (Chen et al., 2024). Three repetitions of the experiment were performed.

### 2.10 Reverse transcription polymerase chain reaction (RT-PCR)

A RNeasy Mini kit (Qiagen, Shanghai, China) was used to extract the total RNA from the nematodes (∼1 mg), which was reverse-transcribed using a cDNA synthesis kit (Bio-Rad Laboratories, Shanghai, China), while SYBR Premix Ex Taq (Takara, Shanghai, China) was used for qRT-PCR. The tba-1 reference protein encoding tubulin was used for relative gene quantification (Wu et al., 2024). The experiment was conducted in triplicate across three groups.

### 2.11 RNA interference

The dsRNA of the target gene was cloned into the L4440 expression vector, after which the vector plasmid was transformed into *E. coli* HT115. NGM plates were fortified with 1 mM IPTG and 50 μg/mL ampicillin. The induced bacteria were inoculated onto these NGM plates and cultured at room temperature for 24 h to allow sufficient dsRNA expression (Timmons, 2006). Prior to the experiment, the PA14-infected worms were cultured on RNAi plates and treated with RA.

### 2.12 Data analysis

The findings are presented as mean ± standard error of the mean (SEM). Statistical differences between data were tested for significance using one-way or two-way analysis of variance (ANOVA). A value of **P < 0.01 represents significant differences. All data were statistically analyzed and plotted using GraphPad Prism 10.1.2.

## 3 Results

### 3.1 RA inhibits the number of PA14 colonies in *C. elegans*

**FIGURE 1:**
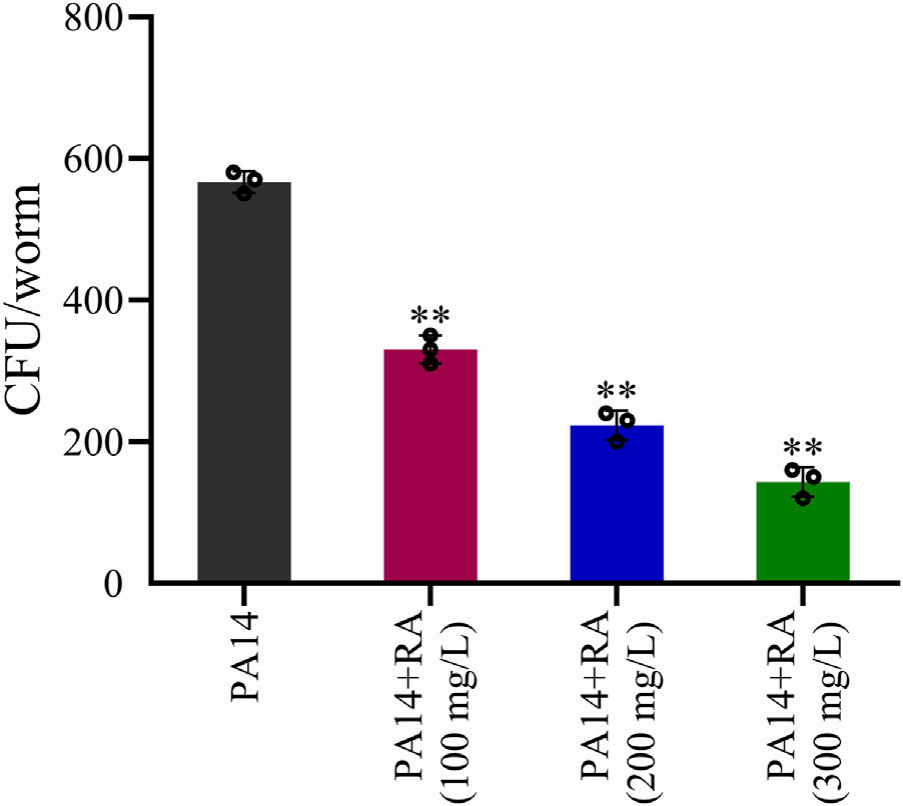
Effect of RA on the number of colonies in *C. elegans.* ^**^ *P* < 0.01 vs. control.

Colony counting indicated that RA significantly reduced the number of colonies in the PA14-infected worms (Figure 1). After treatment with RA at concentrations of 100, 200, and 300 mg/L, the colony count was reduced by 42%, 61%, and 75%, respectively, compared with the PA14 group. These results indicate that as the concentration of RA increases, its inhibitory effect on PA14 is gradually enhanced.

### 3.2 RA extends the lifespan of PA14-infected *C. elegans*

**FIGURE 2:**
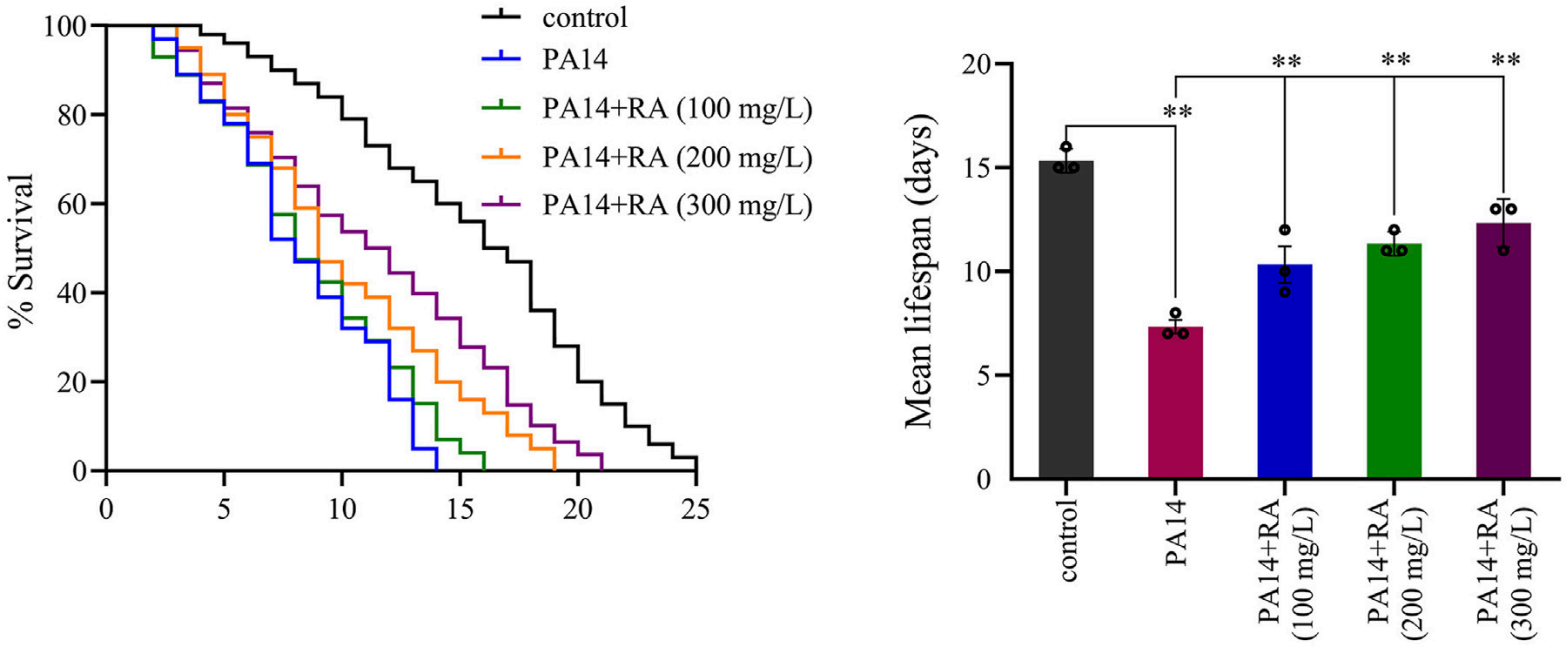
Ability of RA to improve the lifespan of PA14-infected *C. elegans*. ^**^ *P* < 0.01.

Relative to the control group, PA14 infection significantly reduced the lifespan of the worms (Figure 2). Exposure to different RA concentrations extended the lifespan of the PA14-infected worms to varying degrees (Figure 2), with the effect becoming more pronounced as the RA concentration increased.

### 3.3 RA ameliorates the healthy behavior of PA14-infected *C. elegans*

**FIGURE 3:**
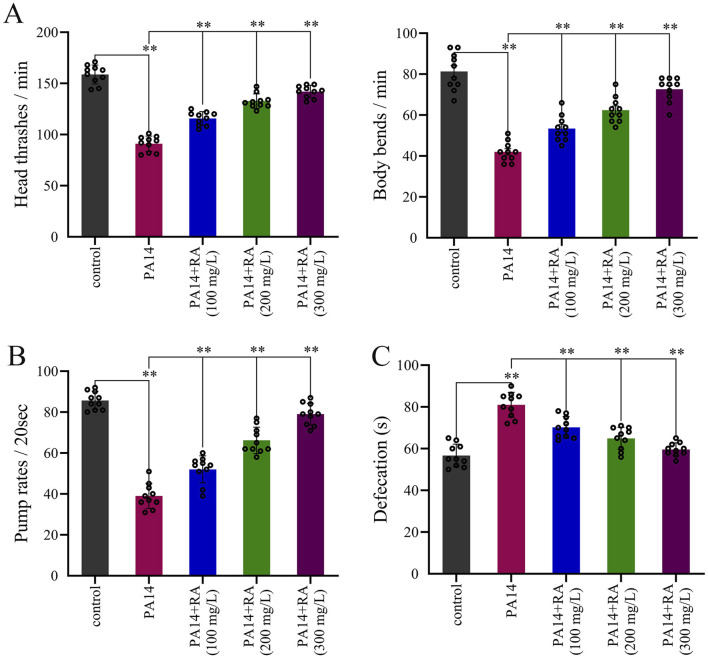
Effect of RA on the healthy behavior of PA14-infected *C. elegans*. **(A)** Impact of RA on the locomotor behavior of the worms after PA14 infection. ^**^ *P* < 0.01. **(B)** Impact of RA on the pharyngeal pumping rates of the worms after PA14 infection. ^**^ *P* < 0.01. **(C)** Effect of RA on the defecation of the worms after PA14 infection. ^**^ *P* < 0.01.

PA14 infection adversely affected the physiological behavior of the worms. Compared to the blank control group, the PA14-infected worms exhibited a significant decrease in head-thrashing, body-bending, and pharyngeal pumping rates (Figures 3A,B), along with a relatively prolonged excretion time (Figure 3C). These results indicated that PA14 impaired the locomotor ability, reduced vitality, and weakened the swallowing and excretion capacity of the worms. Exposure to different RA concentrations alleviated the adverse effect of PA14 on the worms in all three RA-treated groups. Compared to those in the PA14-infected group, the frequencies of head thrashing and body bending, along with the pharyngeal pumping rate, increased significantly (Figures 3A,B), while the excretion cycle was considerably shorter (Figure 3C).

### 3.4 RA reduces lipofuscin and ROS accumulation in PA14-infected *C. elegans*

**FIGURE 4:**
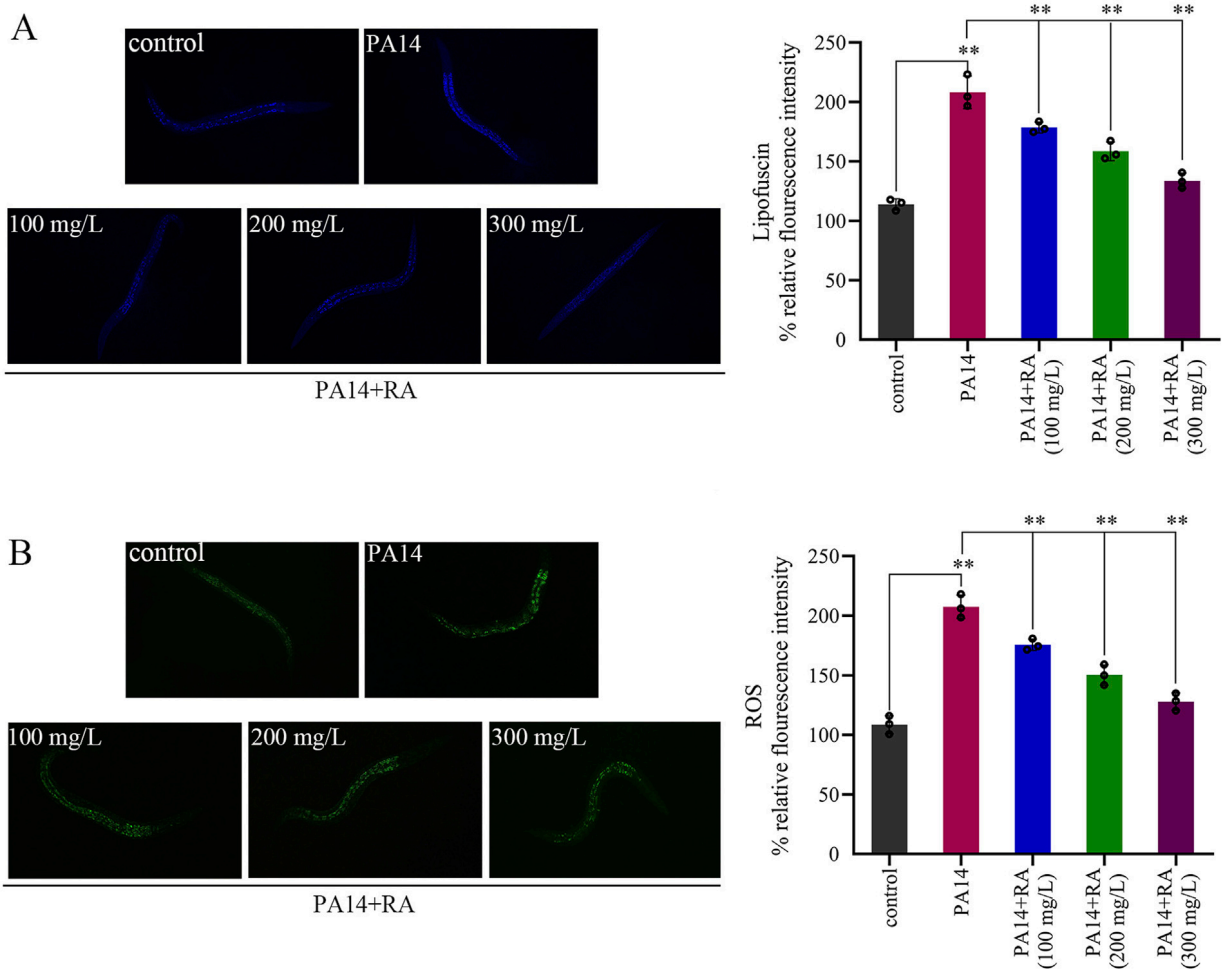
RA modulates lipofuscin and ROS accumulation in PA14-infected *C. elegans*. **(A)** Effect of RA on the lipofuscin level in the PA14-infected worms. ^**^ *P* < 0.01. **(B)** Effect of RA on the ROS level in the PA14-infected worms. ^**^ *P* < 0.01.

Relative to the blank control group, PA14 infection significantly increased the fluorescence intensity of lipofuscin and ROS in the worms, which decreased after RA treatment (Figures 4A,B). These results indicated that PA14 infection induced oxidative damage in the worms since excessive ROS promoted lipofuscin accumulation. Treatment with RA significantly reduced the levels of lipofuscin and ROS.

### 3.5 RA enhances mitochondrial function in PA14-infected *C. elegans*

**FIGURE 5:**
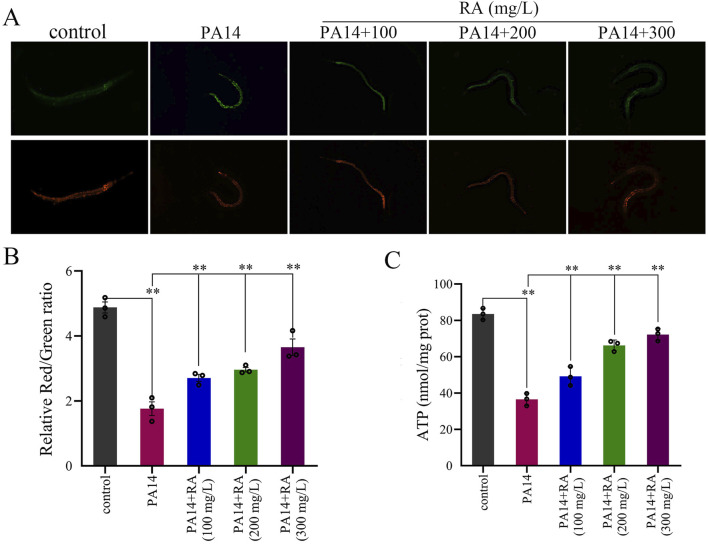
RA modulates mitochondrial function in PA14-infected *C. elegans*. **(A,B)** The effect of RA on the MMP of the PA14-infected worms. ^**^P < 0.01. **(C)** The effect of RA on the ATP of the PA14-infected worms. ^**^ *P* < 0.01.

PA14 caused oxidative damage in the worms. Excessive ROS accumulation impaired the mitochondrial membrane structure and inhibited mitochondrial function (Lei et al., 2013). The MMP measurements were used to further investigate the impact of PA14 on the mitochondria of the worms. The worms infected by PA14 displayed a decrease in the proportion of mitochondria exhibiting high membrane potential (red fluorescence) and an increase in those showing low membrane potential (green fluorescence), reducing the total membrane potential. RA treatment decreased the proportion of mitochondria displaying low membrane potential (green fluorescence) and increased the total membrane potential (Figure 5A).

The experiments demonstrated that a significant decrease in the ATP content accompanied the PA14-induced reduction in the MMP of the worms. Contrarily, RA treatment increased both the ATP level and MMP (Figure 5B), with the overall trend consistent with the MMP results. Additionally, the efficacy of RA was enhanced as its concentration increased, exhibiting a clear dose-dependent effect.

### 3.6 The effect of RA on *mev-1* and *gas-1* in PA14-infected *C. elegans*

**FIGURE 6:**
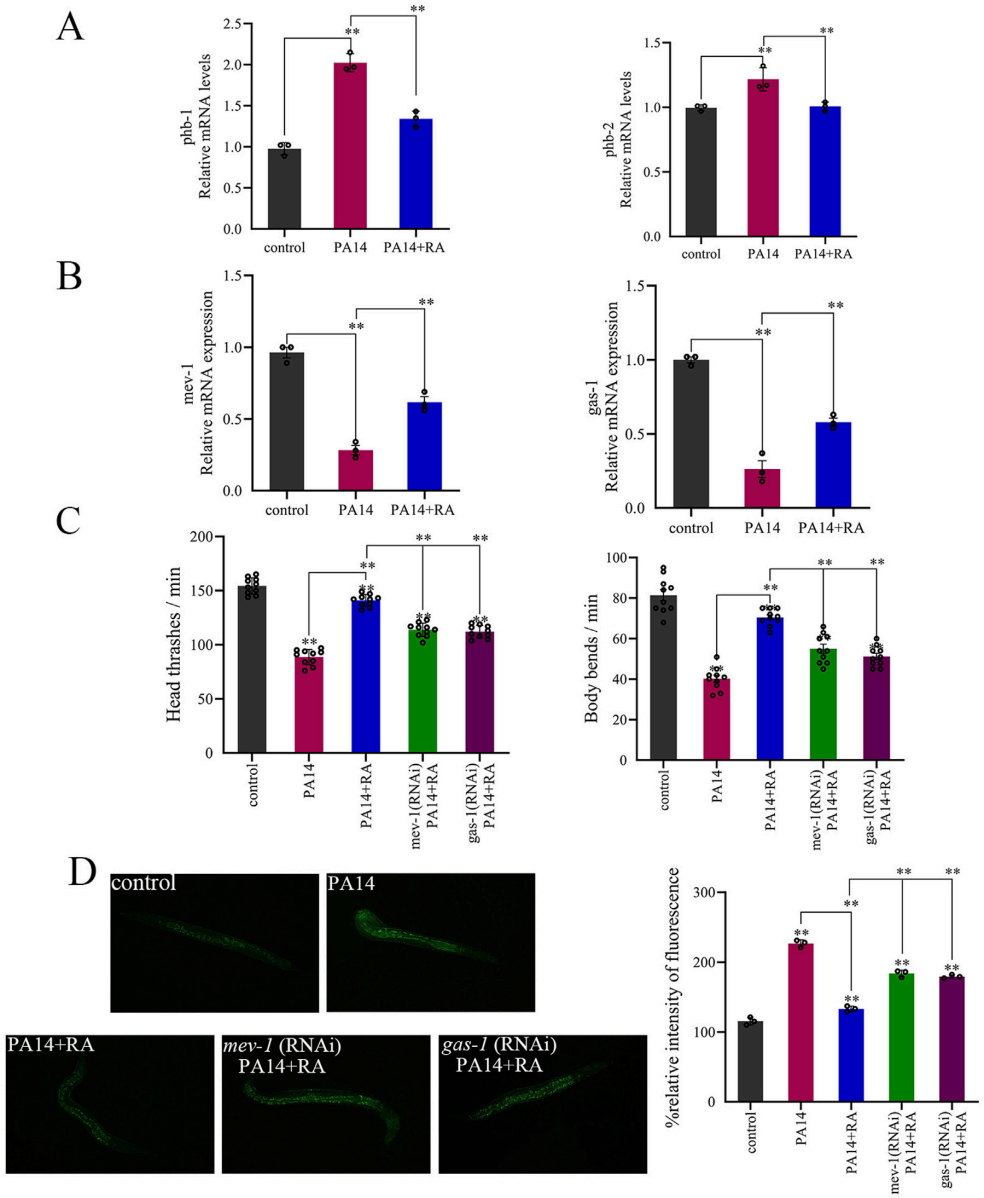
RA regulates the *phb-1*, *phb-2*, *mev-1*, and *gas-1* genes in PA14-infected *C. elegans*. **(A)** *phb-1* and *phb-2* expression after RA treatment **(B)** *mev-1* and *gas-1* expression after RA treatment. Effect of RA on the **(C)** locomotor behavior and **(D)** ROS of the PA14-infected worms with RNAi *mev-1* and *gas-1.* The RA exposure concentration was 300 mg/L. ^**^ *P* < 0.01.

Previous studies have found that *phb-1* and *phb-2* can affect the MMP of *C. elegans* (Qu et al., 2023). Verified in this experiment, PA14 infection of *C. elegans* triggers stress and immune responses, accompanied by upregulated expression levels of *phb-1* and *phb-2* (Figure 6A). After treatment with RA, the expression levels of these two genes decrease and approach the normal level. This indicates that RA combats PA14 infection by regulating mitochondria. The mitochondrial *mev-1* and *gas-1* genes in *C. elegans* are involved in the assembly and function of mitochondrial complexes I and II, respectively (Li et al., 2018; Kayser et al., 2001). The expression levels of *mev-1* and *gas-1* decreased significantly in the PA14-infected worms but were alleviated by RA treatment (Figure 6B). RA treatment reduced the locomotor ability of the worms displaying *mev-1* and *gas-1* RNAi (Figure 6C) while increasing ROS accumulation *in vivo* (Figure 6D).

### 3.7 Evaluation of the safety of RA on *C. elegans*

**FIGURE 7:**
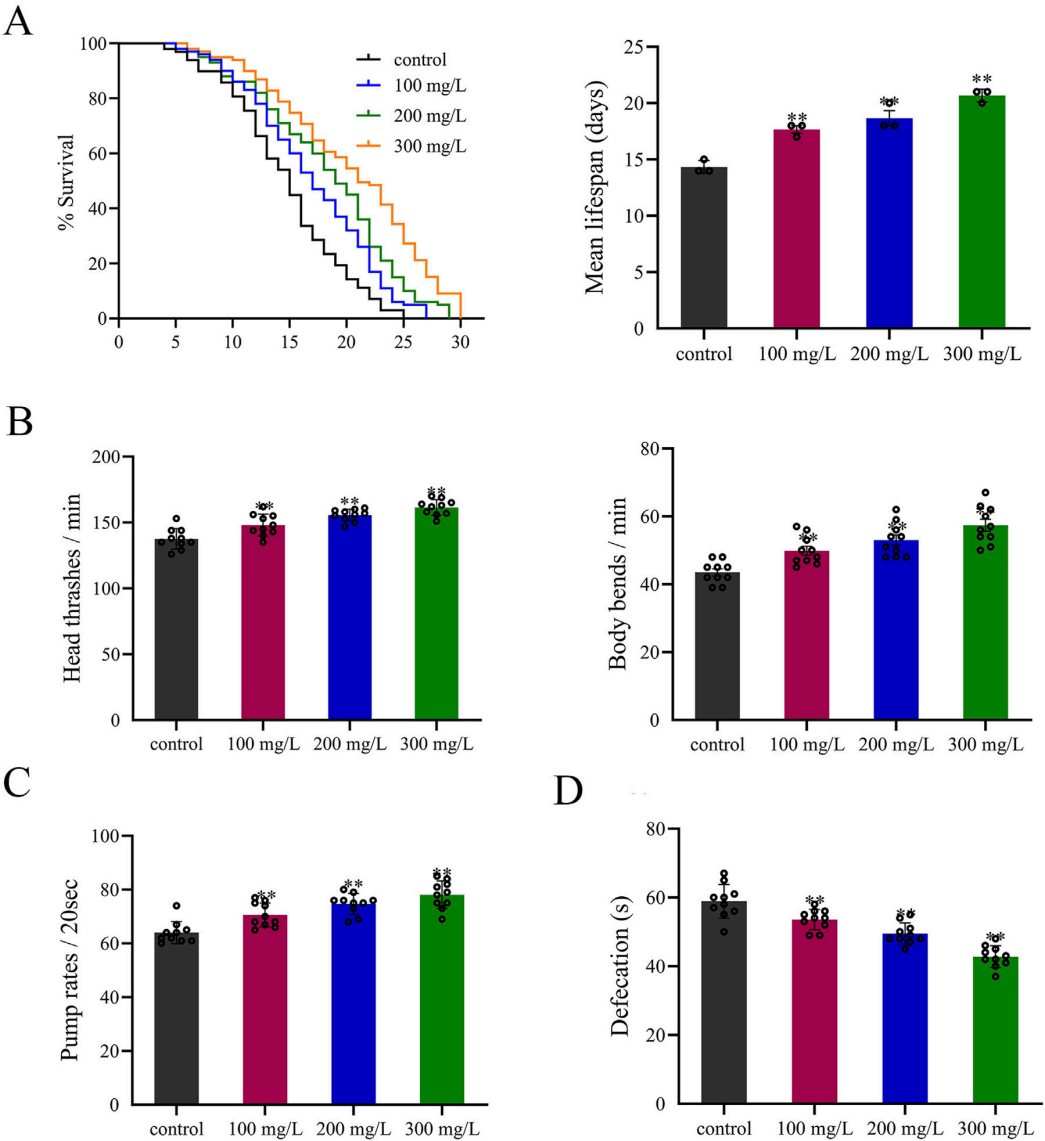
Effects of RA on the **(A)** lifespan, **(B)** locomotor behavior, **(C)** pharyngeal pumping rate, and **(D)** excretion cycle of *C. elegans*. ^**^ *P* < 0.01 vs. control.

When *C. elegans* was treated with RA, the lifespan curves of the nematodes shifted to the right at all three concentrations. In terms of the mean lifespan, a 300 mg/L concentration facilitated the most pronounced extension of the nematode lifespan (Figure 7A). Regarding locomotion, RA significantly enhanced the frequencies of nematode head thrashing and body bending compared to the blank group, showing a dose-dependent effect (Figure 7B). RA also positively affected pharyngeal pumping and excretion of the worms. RA treatment increased the pharyngeal pumping rate and reduced the excretion cycle, exhibiting a similar dose-dependent effect (Figures 7C,D).

## 4 Discussion

*P. aeruginosa* is a widely distributed Gram-negative pathogenic bacterium that causes damage to the ecological environment, such as reducing soil microbial diversity and inducing water eutrophication. It can also infect animals, plants, and humans, leading to diseases such as pneumonia, exacerbating the deterioration of lung function in cystic fibrosis, and even causing death (Wood et al., 2023). RA, a naturally occurring phenolic acid compound, exhibits a range of biological activities, encompassing antibacterial, antioxidant, and anti-inflammatory properties (Guan et al., 2022). However, the specific mechanisms underlying its ability to resist PA14 infections remain unclear.

Numerous studies have demonstrated the effects of RA in the *C*. *elegans* model, and its mechanism of action is closely associated with evolutionarily conserved signaling pathways. RA extends the lifespan of *C. elegans* while maintaining their reproductive capacity and effectively delays age-related phenotypic deterioration; this process is associated with changes in the expression of core regulatory genes in the insulin/insulin-like growth factor signaling pathway and the p38 MAPK pathway (Wang et al., 2012). Antioxidant stress resistance is a core active feature of RA. It enhances the activity of antioxidant enzymes in *C. elegans*, regulates the glutathione redox balance, and strengthens the nematodes' resistance to heat stress and oxidative toxicants (Lin et al., 2019). This effect is directly associated with the activation of the expression of downstream antioxidant-related genes. Additionally, in the field of neuroprotection (Chen et al., 2024), RA can inhibit the abnormal aggregation of pathogenic proteins and reduce neuronal damage in *C. elegans* models of Alzheimer’s disease, Huntington’s disease, and Parkinson’s disease. Its protective effect may be associated with the improvement of oxidative stress status and mitochondrial dysfunction.

This study demonstrated that RA displayed a dose-dependent ameliorative effect on PA14-infected *C*. *elegans* in a concentration range of 100–300 mg/L. Colony counting experiments showed that PA14 infection significantly increased the bacterial number in the bodies of the *C. elegans* worms, while RA treatment substantially reduced the number of colonies (Figure 1). Our findings demonstrated that RA mitigated the adverse implication of PA14 on the worm lifespan, exhibiting a clear dose-dependent relationship. This indicated that RA directly inhibited the colonization ability of PA14 in the host, while a higher RA concentration enhanced the antibacterial effect. This may be because RA disrupts the PA14 cell membrane structure or inhibits its nucleic acid and protein synthesis, consequently reducing intestinal bacterial proliferation (Zhang et al., 2022b).

PA14 infection significantly reduced the nematode lifespan, which was prolonged by RA treatment in a dose-dependent manner. Among the various treatment groups, the median lifespan was most notably extended in the 300 mg/L RA group compared with the infected group (Figure 2). Our findings demonstrated that RA mitigated the adverse implication of PA14 on the worm lifespan, exhibiting a clear dose-dependent relationship. Behavioral analysis further confirmed that RA ameliorated PA14-induced behavioral deficits (Figure 3A). As shown in Figure 3B, a higher RA concentration significantly improved the pharyngeal pumping frequency of the nematodes, which reflected feeding ability (Trojanowski et al., 2016). Furthermore, RA treatment reduced the excretion cycle, an indicator of intestinal function and metabolic capacity (Spanier et al., 2009), suggesting that RA repaired the intestinal function of nematodes and enhanced their metabolic capacity (Figure 3C). The results obtained indicated that the ability of RA to improve the life quality of infected nematodes was clearly dose-dependent, with higher RA concentrations proving to be more effective in restoring their motor, feeding, and intestinal functions.

PA14 infection resulted in significant ROS and lipofuscin (a marker of oxidative damage) accumulation in the nematodes. Experimental results revealed that RA mitigated lipofuscin and ROS aggregation in the PA14-infected worms (Figure 4), alleviated the related oxidative damage, and exhibited a dose–effect relationship. This phenomenon was directly related to the strong antioxidant activity of RA (Villalva et al., 2018), which alleviated the oxidative stress damage induced by PA14 infection through pathways such as scavenging of free radicals and inhibition of lipid peroxidation.

Mitochondria are vital to cellular energy metabolism and are the primary target of oxidative stress-induced damage (Kakkar and Singh, 2007). This study found that the anti-infective effect of RA was closely associated with improved mitochondrial function. Experimental studies have revealed that infection with PA14 impairs mitochondrial function in *C. elegans*. According to experimental results, excessive ROS levels directly attack the mitochondrial membrane structure, leading to a decrease in MMP (Figure 5A). MMP converts the energy generated by the electron transport chain into ATP through chemiosmotic potential (Boyer, 1997); therefore, a decrease in MMP simultaneously results in impaired ATP synthesis and a reduction in ATP levels (Figure 5B). Moreover, surplus ROS also corroborated mitochondrial function impairment, further exacerbating ROS release, creating a vicious cycle of oxidative stress and mitochondrial damage (Gallo et al., 2024). This damage directly affected the physiological functions of *C. elegans*. ATP deficiency reduced muscle contractility (Galimov et al., 2018), which manifested as lower head swing and body bend frequency (Figure 3A). Abnormal mitochondrial function in intestinal cells impaired digestion and excretion (Williams et al., 2022), which decreased the pharyngeal pumping frequency and prolonged the excretion cycle (Figures 3B,C). Furthermore, the accelerated cellular senescence (lipofuscin accumulation) caused by mitochondrial damage, combined with insufficient energy supply, collectively reduced the *C. elegans* lifespan. The experiment revealed that RA improved mitochondrial function via dual mechanisms. First, RA exhibited direct antioxidant effects. It reduced oxidative damage to mitochondrial membranes by decreasing ROS accumulation, consequently increasing the MMP by 58% in the 300 mg/L group (Figure 5A) and elevating ATP synthesis by 65% (Figure 5B). This enhanced mitochondrial energy metabolism, which provided energy for cellular activities. The restoration of mitochondrial function directly reversed the adverse effect of PA14 infection. An increase in the ATP supply restored muscle function, which significantly improved the behavioral indicators, including the frequencies of head swings, body bends, and pharyngeal pumping. Balanced energy metabolism delayed cellular senescence, reduced lipofuscin deposition, and prolonged the *C. elegans* lifespan. The results indicated that mitochondrial function served as the central hub through which RA exerted its multifaceted anti-infective effects. Behavioral improvements, oxidative stress alleviation, and lifespan extension all relied on MMP stability and increased ATP synthesis. This finding highlighted the central role of mitochondria in the host resistance to PA14 infection and provided a clear functional target for the anti-infective mechanism of RA.

The results further confirmed that RA protected mitochondrial function by upregulating the expression levels of *mev-1* and *gas-1*, mitochondrial function-related genes. The *mev-1* gene encodes a key subunit of mitochondrial complex I and is involved in the assembly and functional maintenance of the electron transport chain. The *gas-1* gene regulates the activity of mitochondrial complex II, while both collectively affect the efficiency of mitochondrial oxidative phosphorylation (Li et al., 2018; Kayser et al., 2001). PA14 infection significantly downregulated *mev-1* and *gas-1* expression (Figure 6A), impairing complex I and II functionality, which in turn decreased the synthesis of MMP and ATP. This gene expression downregulation represents a vital molecular mechanism through which PA14 disrupts mitochondrial function.

RA treatment reversed the PA14-induced inhibition of *mev-1* and *gas-1*, consequently upregulating their expression (Figure 6A). The restoration of gene expression directly promoted the assembly and functional activation of mitochondrial complexes, as evidenced by increased MMP and ATP synthesis. These results indicated that RA fundamentally improved mitochondrial function by regulating core mitochondrial genes at the transcriptional level. RNAi experiments further validated the critical role of *mev-1* and *gas-1*. Silencing these two genes significantly attenuated the protective effect of RA on infected nematodes, as reflected by lower ROS accumulation and improved locomotor activity (Figures 6B,C). By upregulating the expression levels of the *mev-1* and *gas-1* mitochondria-related genes, RA promoted the assembly and functional activation of mitochondrial complexes I and II, enhanced MMP and ATP synthesis, and reduced ROS accumulation, consequently disrupting the “gene downregulation–mitochondrial dysfunction–oxidative stress” vicious cycle triggered by PA14 infection. Ultimately, this inhibited bacterial proliferation, improved behavioral function, alleviated oxidative damage, and extended lifespan. These findings confirmed that *mev-1* and *gas-1* represented key targets for RA action, while their upregulated expression formed the molecular basis for the enhancement of mitochondrial function and anti-infection properties by RA.

In this research, *C. elegans* was adopted as a model organism to systematically investigate the role of RA in combating PA14 infection and the underlying molecular mechanisms. Within a specific dose range, RA prolonged the lifespan of *C. elegans* and enhanced their locomotor capacity, feeding ability, and intestinal function. The results showed that RA inhibited PA14 infection in a dose-dependent manner, which was closely associated with enhanced antioxidant activity, improved mitochondrial function, and regulation of mitochondria-related genes. In addition to the antioxidative and mitochondrial protective mechanisms highlighted in this study, the ability of RA to inhibit PA14 infection may also stem from its role in enhancing host immune response. Future studies can further explore whether RA enhances host resistance to pathogens by regulating immune-related genes.

*P. aeruginosa* displays strong environmental adaptability, easily surviving and reproducing in humid environments, and demonstrating considerable potential for causing pollution. RA, a naturally occurring compound, is present in diverse plant species, offering advantages such as low toxicity, easy extraction, and multi-target properties. Genes of *C. elegans* share high sequence conservation with human genes. In experiments, the *C. elegans* genes *phb-1*, *phb-2*, *mev-1*, and *gas-1* correspond to the human homologous genes *PHB-1* (Nijtmans et al., 2002), *PHB-2* (Nijtmans et al., 2002), *SDHC* (Ishii et al., 2011), and *NDUFS2* (Pujol et al., 2013), respectively. The mechanism by which RA combats PA14 infection by regulating these four genes in the *C. elegans* model can provide target references for the treatment of human diseases caused by PA infection.

RA naturally exhibits extremely low oral bioavailability (<10%), mainly limited by poor lipophilicity, degradation by intestinal flora, and hepatic first-pass metabolism (Kang et al., 2021). Nearly complete absorption can be achieved via direct intravenous/intraperitoneal administration; transdermal administration requires penetration enhancers to overcome the skin barrier; nasal or pulmonary nebulization enables targeted delivery to the brain or lungs (Hitl et al., 2021); for oral administration, technologies such as self-microemulsifying systems, esterified derivatives, or co-administration with flavonoids/piperine can increase its bioavailability by 1.7–9 folds, meeting diverse clinical needs (Kang et al., 2021). This study provides a theoretical basis for the use of RA in the treatment of *P. aeruginosa* infections. RA can serve as an adjuvant for the clinical treatment of *P. aeruginosa* infections; however, further research is still needed to explore ways to improve its bioavailability and develop more dosage forms, thereby enabling it to function as a primary therapeutic drug against *P. aeruginosa* infections.

## 5 Conclusion

The *C. elegans* animal model showed that PA14 infection caused varying degrees of damage in the worms, ranging from organismal phenotypes to mitochondrial dysfunction. Treatment with different RA concentrations ranging between 100 mg/L and 300 mg/L exhibited a dose-dependent effect on the lifespan, locomotion, swallowing, and excretion of the infected worms. Additionally, RA reduced lipofuscin accumulation, counteracted PA14-induced ROS production, and significantly alleviated oxidative stress. Furthermore, RA mitigated PA14-induced mitochondrial dysfunction by regulating the *mev-1* and *gas-1* mitochondria-related genes, consequently enhancing the MMP and ATP synthesis. The high toxicity and infectivity of PA14 present substantial threats to both the environment and organisms. Therefore, this research demonstrated that RA inhibited the damage caused by PA14 infection, highlighting its potential as a natural compound for addressing PA14 infection.

## Data Availability

The original data presented in the study are included in the article; further inquiries can be directed to the corresponding author.
