# Supplementary material for: The protective effects and mechanisms of rosmarinic acid against Pseudomonas aeruginosa infection in Caenorhabditis elegans
Source: Front Pharmacol. 2025 Dec 2;16:1701885. doi: 10.3389/fphar.2025.1701885 (PMC12705596; doi:10.3389/fphar.2025.1701885)
Supplement: Supplementary file 1 [file DataSheet1.doc]

**Supporting Information:**


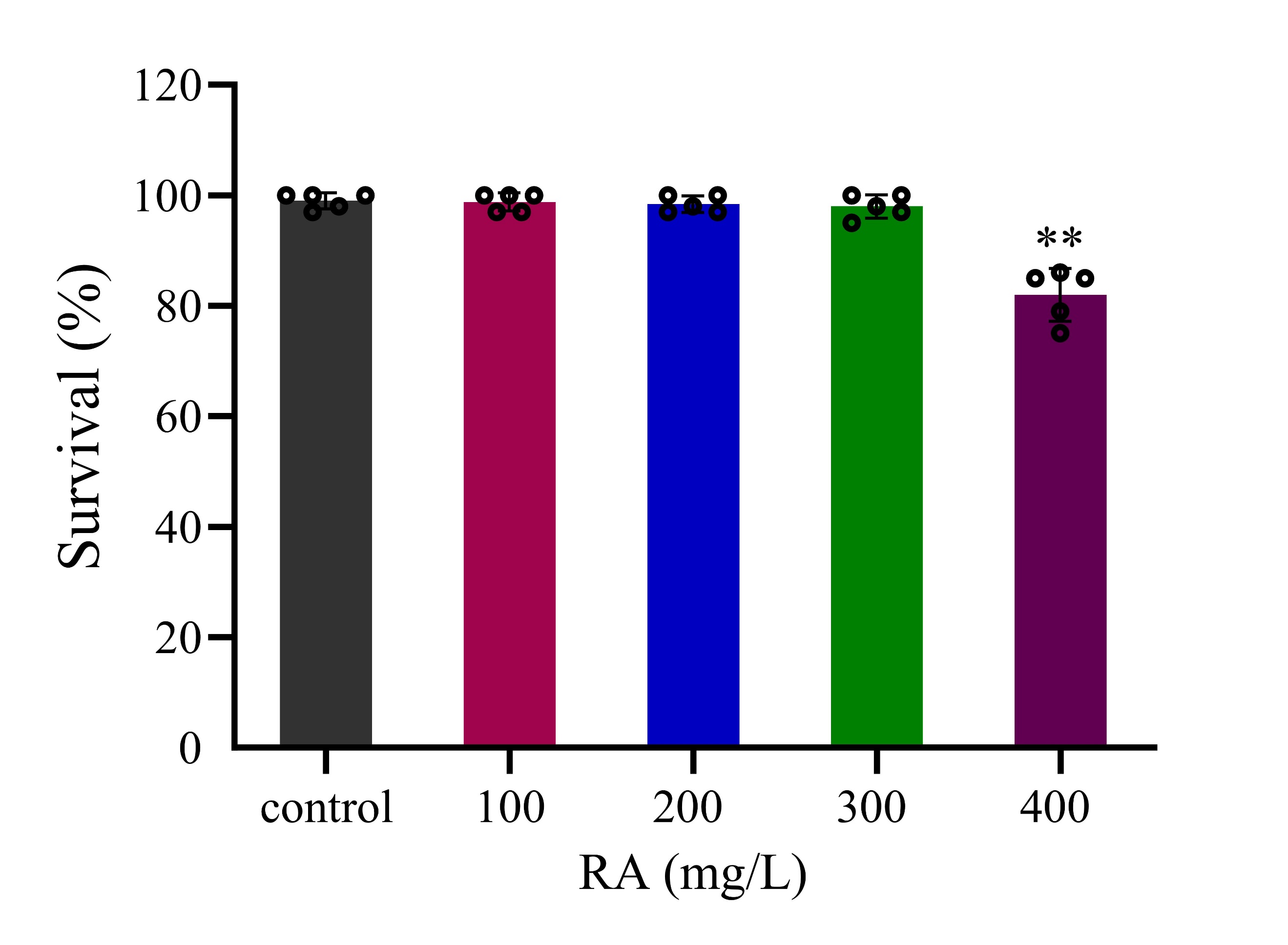


**Figure S1.** The effect of different concentrations of RA on the survival rate of Caenorhabditis elegans. ^**^*P <* 0.01

**Table S1.** Primer information for qRT-PCR in *C. elegans*

| Gene | Forward primer (5’-3’) | Reverse primer (5’-3’) |
| --- | --- | --- |
| *mev-1* | TCGGCTATTCTTGCTCTCGC | ACGAGAATTGTCGAGGGCTG |
| *gas-1* | TCTCAACTTCGGACCACAGC | CAACAGCCAAAGACCAAGCC |
| *act-1* | GCTGAAGATGACCCAGATCA | CGTACAGGGATAGCAACAGG |
